# How clonal are clones? A quest for loss of heterozygosity during asexual reproduction in *Daphnia magna*

**DOI:** 10.1111/jeb.13443

**Published:** 2019-04-10

**Authors:** Marinela Dukić, Daniel Berner, Christoph R. Haag, Dieter Ebert

**Affiliations:** ^1^ Zoological Institute University of Basel Basel Switzerland; ^2^ Department of Cell and Developmental Biology John Innes Centre Norwich Research Park Norwich UK; ^3^ Centre d'Ecologie Fonctionnelle et Evolutive—CEFE UMR 5175 CNRS—Université de Montpellier—Université Paul‐Valéry Montpellier—EPHE, campus CNRS Montpellier France; ^4^ Department of Biology, Ecology and Evolution University of Fribourg Fribourg Switzerland

**Keywords:** ameiotic recombination, apomixis, loss of complementation, RAD‐sequencing

## Abstract

Due to the lack of recombination, asexual organisms are predicted to accumulate mutations and show high levels of within‐individual allelic divergence (heterozygosity); however, empirical evidence for this prediction is largely missing. Instead, evidence of genome homogenization during asexual reproduction is accumulating. Ameiotic crossover recombination is a mechanism that could lead to long genomic stretches of loss of heterozygosity (LOH) and unmasking of mutations that have little or no effect in heterozygous state. Therefore, LOH might be an important force for inducing variation among asexual offspring and may contribute to the limited longevity of asexual lineages. To investigate the genetic consequences of asexuality, here we used high‐throughput sequencing of *Daphnia magna* for assessing the rate of LOH over a single generation of asexual reproduction. Comparing parthenogenetic daughters with their mothers at several thousand genetic markers generated by restriction site‐associated DNA (RAD) sequencing resulted in high LOH rate estimation that largely overlapped with our estimates for the error rate. To distinguish these two, we Sanger re‐sequenced the top 17 candidate RAD‐loci for LOH, and all of them proved to be false positives. Hence, even though we cannot exclude the possibility that short stretches of LOH occur in genomic regions not covered by our markers, we conclude that LOH does not occur frequently during asexual reproduction in *D. magna* and ameiotic crossovers are very rare or absent. This finding suggests that clonal lineages of *D. magna* will remain genetically homogeneous at least over time periods typically relevant for experimental work.

## 1 INTRODUCTION

In eukaryotes, asexuality is a state derived from sexual reproduction and this transition has occurred many times independently throughout metazoan evolution (Simon, Delmotte, Rispe, & Crease, 2003). Parthenogenesis is an umbrella term for asexual reproduction in animals where offspring develop from unfertilized eggs but the mechanisms through which the parental ploidy can be retained are highly diverse (Archetti, 2010; Schön, Martens, & van Dijk, 2009; Suomalainen, Saura, & Lokki, 1987). Based on the presence or absence of meiosis, there are two main types of parthenogenesis: automixis and apomixis. In automictic parthenogenesis (automixis), normal meiosis with chromosome reduction takes place and the somatic ploidy is restored by duplication or fusion of products of the same meiosis. In apomictic parthenogenesis (apomixis), meiosis and recombination are considered to be absent, and such ameiotic conditions are assumed to result in clonal offspring. Since apomixis is the most common type of asexual reproduction in nature (Archetti, 2010; Lehtonen, Jennions, & Kokko, 2012; Suomalainen et al., 1987), asexuality is used almost as a synonym for apomixis. Thus, it is widely accepted that asexual reproduction leads to the production of clonal offspring with rare mutations being the only source of genetic variation. This assumption has played an important role in evolutionary biology, especially in models aiming to explain the evolutionary advantage of sex. Yet, despite its important consequences, little is known about the genetic consequences of apomictic reproduction in animals.

In the complete absence of recombination during ameiotic reproduction, two alleles are expected to accumulate mutations independently over time, generating high levels of allelic divergence among asexual lineages (Mark Welch & Meselson, 2000). However, asexual lineages studied so far do not show such an effect, but rather the opposite (Birky, 2004; Hartfield, 2015). For example, Darwinuloid ostracods and oribatid mites show lower levels of allelic divergence than their sexual counterparts (Schaefer et al., 2006; Schon & Martens, 2003), suggesting the presence of cryptic genome homogenization processes within these asexual lineages.

Several mechanisms for allelic convergence were proposed (Birky, 2004; Flot et al., 2013; Schaefer et al., 2006), and homology‐based DNA double‐strand break (DSB) repair is a common factor among all of them (i.e. homologous recombination). Depending on the exact mechanisms employed, recombination in apomixis can result in long or short tracts of loss of heterozygosity (LOH). Reciprocal crossover (CO) recombination will lead to long stretches of LOH (assuming frequent co‐segregation of recombined and nonrecombined homologous chromatids), whereas nonreciprocal exchange results in the homozygosity of short DNA tracts (gene conversions), restricting LOH to few hundred base pairs (bp).

Even though CO recombination is usually associated with the meiotic prophase I, evidence of COs under ameiotic conditions comes from studies of mitotic recombination in fungi or somatic cells. In *Saccharomyces cerevisiae*,* Aspergillus niger* and *Candida albicans,* rates of LOH caused by mitotic COs range from 10^−2^ to 10^−7^ per locus per generation (Debets, Swart, Hoekstra, & Bos, 1993; Forche et al., 2011; Mandegar & Otto, 2007). However, in multicellular eukaryotes the situation is more complex. Since asexuality is derived from sexual reproduction, mechanistically, apomixis is more likely to be a modified form of meiosis rather than mitosis. The most common modification is the suppression of the first (reductional) meiotic division which includes homologue pairing and recombination (Archetti, 2010). Since the first meiotic division is suppressed, reduction in the number of chromosomes is not taking place and only sister chromatids separate in a mitotic‐like process (second meiotic division). However, reductional division does not have to be suppressed completely and the remnants of meiosis I have been described in apomictic organisms (Suomalainen et al., 1987).

Karyological studies on apomictic dandelions (van Baarlen, van Dijk, Hoekstra, & de Jong, 2000) and weevils (Rozek, Lachowska, Holecovà, & Kajtoch, 2009) demonstrated pairing of chromosomes and the formation of structures resembling chiasmata (cytological indication of COs). Another compelling evidence of meiotic vestiges during apomixis comes from the study of parthenogenetic mechanisms in *Daphnia pulex*. Hiruta, Nishida, and Tochinai (2010) showed that the diploidy in *D. pulex* is maintained by meiotic arrest at an early anaphase I. Thus, meiosis I is not suppressed, but aborted before separation of homologues takes place. The observed mechanism includes the formation of bivalents in prophase I, implying the opportunity for CO recombination to occur. However, the chiasmata were not observed, which is not surprising given the small size of *Daphnia* chromosomes (Hiruta et al., 2010).

Consistent with the hypothesis of CO occurrence during asexual reproduction, high rates of LOH were reported in mutation‐accumulation lines of *D. pulex* and *Daphnia obtusa* (Omilian, Cristescu, Dudycha, & Lynch, 2006; Xu, Omilian, & Cristescu, 2011). Analysing initially heterozygous microsatellite markers after more than hundred generations of parthenogenetic mutation accumulation, Xu et al. (2011) estimated the rate of ameiotic recombination in *D. pulex* as 3.3 × 10^−5^ per locus per generation. Markers showing LOH clustered together into long stretches of LOH or were confined to the chromosomal tips suggesting that LOH has been caused by CO recombination. More recently, two additional studies (Flynn, Chain, Schoen, & Cristescu, 2017; Keith et al., 2015) using the whole‐genome sequencing of mutation‐accumulation lines of *D. pulex* have reported similar rates of LOH. However, the pattern of LOH differed substantially among studies, and the mechanisms leading to LOH remain elusive. An important issue that arises from the use of mutation‐accumulation lines is potential selection against LOH because it may unmask recessive deleterious mutations. This would prevent or retard the propagation of asexual lines, leading to underestimated values of LOH rates. Indeed, mutation‐accumulation lines from the afore mentioned studies experienced high levels of mortality, forcing authors to use back‐ups in 6%–20% of transfers, thus increasing the opportunity for selection (Flynn et al., 2017; Omilian et al., 2006; Xu et al., 2011).

**Figure 1:**
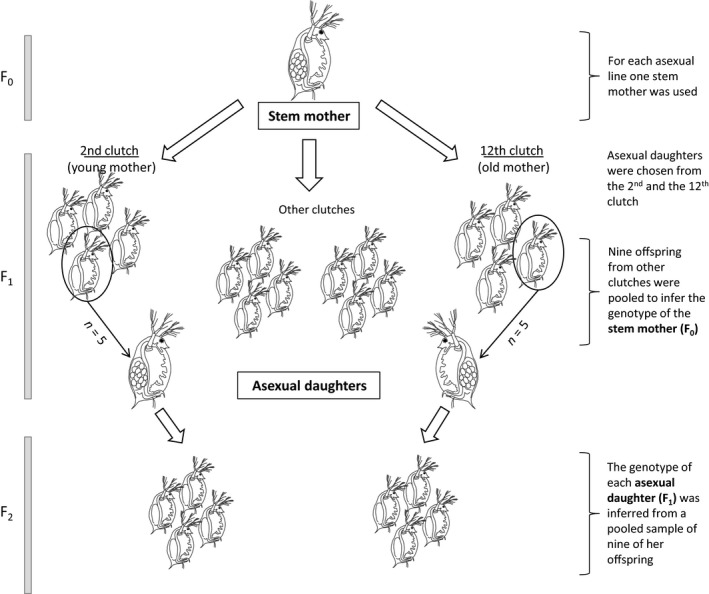
Schematic diagram of the experimental design and sampling procedure. The experiment was replicated for four different clonal lines of *Daphnia magna* and encompassed three generations of asexual reproduction (F_0_–F_2_). Empty arrows indicate generational transition and the production of all‐female clutches. Circles with black arrows indicate five randomly chosen asexual daughters from the first asexual generation (F_1_), produced by young or old stem mother (2nd or 12th clutch, *n* = 5) that were subsequently screened for loss of heterozygosity (LOH). A pool of nine F_1_ females from other clutches was used for genotyping the stem mother (F_0_). A pool of nine F_2_ females was used for genotyping each of the F_1_ asexual daughter (F_1_)

In the current study, we investigated whether LOH due to ameiotic recombination can be detected in a related species, *Daphnia magna*. Asexual reproduction of *D. magna* is described as apomixis with the extrusion of the polar body (Zaffagnini, 1987; but see also Svendsen et al., 2015), suggesting a process derived from meiosis. However, the detailed mechanisms of apomixis in *D. magna* are not well studied and we assume diploidy is maintained by the abortion of the first meiotic division as it was described for *D. pulex* (Hiruta et al., 2010). To assess the rate of LOH with minimal selection, we sought to address this question by examining a single generation of asexual reproduction using several thousand markers generated by restriction site‐associated DNA (RAD) sequencing (Baird et al., 2008). Moreover, since the nuclear genomes become inherently unstable during an organisms senescence (McMurray & Gottschling, 2003), we analysed asexual daughters from young and old mothers (Figure 1) to assess whether the mother's age has any impact on the fidelity of apomixis.

## 2 MATERIALS AND METHODS

### 2.1 Experimental design

*Daphnia magna* is a cyclical parthenogen; that is, it can switch between sexual and asexual reproduction mainly in response to environmental conditions. Laboratory iso‐female cultures are hereafter referred as lines, since they were initiated by a single female and propagated under conditions of continuous asexual (clonal) reproduction. For our experiment, we haphazardly selected four individual females (“stem mothers,” Figure 1) originating from four different lines. The IXF1 line is an inter‐population F1 hybrid, asexually maintained since 2006. The lines RM1–02 and RM1–30 are two distinct clones obtained from a natural population in Moscow Zoo (Russia) that were maintained asexually in laboratory settings for 6 months prior to the start of the experiment. The stem mother of the forth line was hatched from a sexually produced resting egg that was collected from the Aegelsee pond near Frauenfeld (Switzerland), and it served as a founder female of the CH‐H‐876 line. Each stem mother was cultured to produce 13 consecutive clutches of all‐female offspring. Five randomly chosen daughters from the second clutch (young mothers) and five daughters from the 12th clutch (old mothers) were designated as “asexual daughters” (female F1 offspring of stem mothers, Figure 1) and subsequently screened for LOH. In total (across the four lines), we thus investigated LOH in 40 F1 offspring (10 per line) or for a total of 400 chromosomes (*n* = 10 in *D. magna*). Throughout the experiment, animals were kept individually in 100‐ml beakers filled with artificial *Daphnia* medium (Klüttgen, Dülmer, Engels, & Ratte, 1994) at 20°C with 16‐hr light/8‐hr dark cycle and fed with fresh, chemostat‐grown, unicellular algae *Scenedesmus* sp. (five million cells per individual per day). This provided a stress‐free environment since we wanted to minimize the possibility of environmentally induced LOH.

Since we were unable to obtain a sufficient amount of DNA from single individuals for RAD‐sequencing, we used genomic DNA extracted from a pool of nine offspring of a given individual. That is, the asexual F1 daughters of the stem mothers were grown to adulthood and nine offspring of these individuals (i.e. asexual F2 individuals) were pooled to reconstruct the genotype of the F1. Likewise, the genotypes of the stem mothers were inferred from pools of their F1 offspring (from clutches not otherwise used in the experiment). Although each of these individuals might have additional LOH events with respect to their mother (whose genotype we wanted to infer), we assume that each individual would show LOH at different locations in the genome. Thus, by pooling nine individuals, these additional LOH events would not show up. However, any LOH event that occurred between the stem mothers and a given F1 asexual daughter would be present in all asexual F2 daughters of this F1 daughter and hence would be detectable in our pooled sample of nine of these F2 individuals. In addition, to estimate error rates in genotyping RAD‐loci, each stem mother was sequenced twice, that is, using two independent pools of nine F1 offspring.

Prior to sampling, all individuals were cleaned by an antibiotic‐starvation treatment to minimize algal and bacterial contamination of genomic DNA. More precisely, animals were kept for 3 days in a medium containing Ampicillin (Sigma), Streptomycin (Sigma) and Tetracycline (Sigma) at a concentration of 50 mg/L each and transferred daily to fresh antibiotic medium. To enforce the evacuation of gut content, a small amount of superfine Sephadex ^®^ G‐25 (Sigma‐Aldrich) was added frequently to the antibiotic medium. Animals with clear intestines were sampled, and genomic DNA was extracted using the DNeasy Blood and Tissue kit (Qiagen) with minor modifications and an inclusion of RNaseA (100 mg/ml; Sigma) digestion step.

### 2.2 RAD library preparation and sequencing

We prepared libraries for RAD‐sequencing (Baird et al., 2008) adopting the protocol of Etter, Bassham, Hohenlohe, Johnson, and Cresko (2011) with modifications according to Roesti, Moser, and Berner (2013). Specifically, 1 μg of genomic DNA from each sample (pooled genomic DNA of nine individuals) was digested with the *Pst1* HF restriction enzyme (NEB) in 50 μl reaction volume for 90 min at 37°C and then heat‐inactivated following the manufacturer's manual. P1 sequencing adapters (5 μl of 100 nM stock) containing a unique 5‐bp barcode were ligated to each sample using T4 DNA‐ligase (NEB, 0.5 μl of 2,000,000 units/ml stock) in a 60 μl volume for 45 min at room temperature. The reaction was then heat‐inactivated for 20 min at 65°C. The total of 48 samples (four *Daphnia* lines, each represented with two mother samples and 10 daughter samples) were combined into two pools (each containing two *Daphnia* lines, i.e. 24 samples) and sheared using a Bioruptor (Diagenode). DNA fragments in a range of 250–500 bp were selected using agarose gel electrophoresis (1.25%, 0.5× TBE), purified and blunt‐ended (Quick Blunting Kit, NEB). dA‐overhangs were added to the DNA fragments using the Klenow fragment exo^−^ (NEB), followed by P2 adapter ligation (1 μl from 10 mM stock). Products were purified, and PCR amplification was done using the Phusion High‐Fidelity DNA polymerase. To minimize the probability of PCR errors, master mixes for each library were divided into eight separate 12.5 μl reactions for amplification (30 s at 98°C, 17 cycles of 98°C 10 s, 65°C 30 s, 72°C 30 s, then a final extension for 5 min at 72°C). Prepared libraries were sequenced on separate Illumina HiSeq2000 lanes using 100‐bp single‐end sequencing (Quantitative Genomics Facility service platform, Deep Sequencing Unit Department of Biosystems Science and Engineering, ETH Zurich in Basel, Switzerland).

### 2.3 Bioinformatics analysis

The quality of each sequenced library was assessed using FastQC (Babraham Bioinformatics, The Babraham Institute). A custom R script was used to sort reads into individual samples according to the unique barcodes. We discarded sequences containing ambiguous bases and reads that did not feature a valid *Pst1* restriction site. Reads were aligned to the *D. magna* genome (v2.4; Daphnia Genomic Consortium, WFleaBase) using Novoalign v2.07 (http://novocraft.com) tolerating on average one high‐quality mismatch per 12 bases. Only loci aligning to unique genomic locations were considered in further analyses. The average coverage obtained per individual per RAD‐locus was 115x (*SD* = 17.9) for IXF1, 134x (*SD* = 28.3) for RM1–30, 146x (*SD* = 16.5) for RM1–02, and 136x (*SD* = 16.4) for the CH‐H‐876 line, respectively. On average, 24,392 unique RAD‐loci were obtained for each line.

Single nucleotide polymorphism (SNP) calling and genotyping was done using custom R scripts, benefiting from Bioconductor packages Biostrings and Rsamtools (Gentleman et al., 2004). Even though only uniquely aligned loci were considered in our analysis, we also excluded loci with excessive coverage (three times higher coverage than the overall mean for a given individual) to avoid repetitive sequences that are not represented in the *D. magna* reference genome v2.4. The minimum coverage required for a locus to be assigned as a diploid in each individual was three times lower than the estimated mean. We called homozygous genotype when a locus showed only one haplotype with a read count greater than the threshold for calling a diploid locus, or when the second haplotype occurred in less than three copies (an ad hoc criterion to allow for sequencing error in Illumina generated data). A heterozygous locus was called when the total coverage exceeded the threshold for calling a diploid locus and the rarer haplotype was found in a proportion of more than 25%. Highly polymorphic loci (more than three SNPs) were excluded from downstream analyses. Pooling separate PCR reactions in library preparation steps, samples sequenced at high coverage and stringent filtering criteria in bioinformatic analysis, all strongly limited the possibility for false heterozygous calls.

An error rate was estimated by comparing RAD‐loci from the two samples representing the stem mother of a given line. These two samples served as replicates and should be identical except for errors due to library preparation (e.g. PCR errors), sequencing, SNP calling, and genotype calling. The error rate was calculated by dividing the number of detected differences by the number of comparable loci (i.e. successfully sequenced loci in both stem mother samples; number of RAD‐loci used for the estimation of genotyping error rate in Table 1).

**Table 1:** Summary of LOH information in *Daphnia magna* estimated from RAD‐sequencing data

Line (stem mother)	IXF1	RM1–30	RM1–02	CH‐H‐876
Number of asexual daughters	10	10	10	10
Number of RAD‐loci used for the estimation of genotyping error rate	22,814	23,060	26,738	24,957
Genotyping error rate (per RAD‐locus)	0.00447	0.00221	0	0.00020
Number of informative RAD‐loci (heterozygous in stem mother)	7,684	4,840	6,526	5,644
Number of RAD‐markers	4,303	2,930	5,409	4,785
Total number of LOH events detected bioinformatically	204	90	2	1
Rate of LOH (locus^−1^ generation^−1^)	0.004741	0.003072	0.000037	0.000021
Number of LOH events tested by Sanger sequencing	6	8	2	1
Number of LOH events confirmed by Sanger sequencing	0	0	0	0

*Notes*

The number of RAD‐loci used for the estimation of genotyping error rate is the number of all RAD‐loci that were successfully sequenced and genotyped in both stem mother samples. RAD‐markers are informative (heterozygous) RAD‐loci that were retained after filtering and further assessed for LOH in each asexual daughter. Filtering procedures are described in section 2.

LOH: loss of heterozygosity; RAD: restriction site‐associated DNA.

Analysis of LOH was based on assessing whether the heterozygosity detected in stem mothers is retained in five asexual daughters from the second and the 12th clutches. Thus, only RAD‐loci that were heterozygous in stem mothers were informative for this analysis (total number of informative loci, Table 1). To minimize possible biases of RAD‐sequencing (Catchen, Amores, Hohenlohe, Cresko, & Postlethwait, 2011), only loci that were informative in stem mothers and successfully genotyped in at least eight of 10 asexual daughters were considered as markers for estimating the rate of LOH (number of markers, Table 1).

The rate of LOH *λ* (per locus per generation, Table 1) for each line was calculated following Omilian et al. (2006) *λ* = *h/*(*L × i × T*), where *h* is the total number of LOH events observed, *L* is the number of parthenogenetic events analysed (for each line, we inspected 10 daughters, *L *=* *10) and *i* is the number of markers. In all cases, the number of generations (*T*) was one.

Fourteen markers showing LOH in seventeen daughters were selected and re‐sequenced using Sanger sequencing to provide an independent test of LOH at these loci. Primers were designed using Primer3Plus (Untergasser et al., 2012) based on the *D. magna* reference genome sequence, capturing approximately 100 bp flanking the RAD‐locus on either sides. Re‐sequenced loci and the primers used are listed in Supporting Information Table S1. Each locus was re‐sequenced in the daughter showing LOH, one of the daughters that retained maternal heterozygosity and both stem mother samples. Obtained DNA sequence electropherograms were analysed using CodonCode Aligner software v3.7.1.

## 3 RESULTS

Since we were testing for a rare event of LOH during asexual reproduction, we first wanted to estimate the accuracy of our methodology by estimating the error rates based on the two replicated samples of each of the stem mothers. Stem mother samples of the IXF1 and the RM1–30 line, which were sequenced as a part of the same sequencing library, showed genotype (homozygote/heterozygote) inconsistencies in 0.44% and 0.22% of loci, respectively. No inconsistencies were observed between stem mother samples of the RM1–02, whereas stem mother samples of the CH‐H‐876 line, which were part of the same library as the RM1–02 samples, showed inconsistencies in 0.02% of loci that were successfully sequenced in both samples. Thus, the error rate estimates for *D. magna* lines pooled into the first sequencing library were at least one order of magnitude higher than the error rates for lines that were part of the second sequencing library, indicating the presence of a “library effect” in our data (Table 1).

As expected, the IXF1 line had the highest level of heterozygosity since it is an inter‐population hybrid (total number of informative loci, Table 1). Overall, 33% of RAD‐loci were heterozygous in stem mothers of the IXF1 line, 21% in the RM1–30 line, 24% in the RM1–02 line and 23% in the CH‐H‐876 line. Among heterozygous loci, only those that were successfully genotyped in at least eight of the 10 of daughter samples were used as markers for the investigation of LOH events (see below). In total, LOH was assessed at 4,303 marker loci obtained for the IXF1 line, 2,930 markers for the RM1–30 line, 5,409 markers for the RM1–02 line and 4,785 markers for the CH‐H‐876 line (Table 1).

For each marker locus, we searched instances where asexual daughters became homozygous (or hemizygous) for nucleotide sites that were heterozygous in the mother and these instances are referred to as LOH events. We considered only instances in which all heterozygous sites within a given RAD‐marker (up to three) became homozygous. In total, across all four lines inspected, we found 297 putative LOH events. Two hundred and four LOH events (Table 1) were detected for 192 markers in the IXF1 line (eight markers showing LOH in multiple daughters), and the number of detected LOH events for each asexual daughter ranged from one to 99. In the IXF1 line, three times more LOH events were detected in daughters from the second clutch than daughters from the 12th clutch (156 and 48, respectively). In the RM1–30 line, 53 LOH events were detected in the second clutch daughters and 37 LOH events were found in the 12th clutch daughters, summing up to the total of 90 LOH events (Table 1) for 86 markers (three markers showing LOH in more than one daughter). Two LOH events were detected for two markers in the RM1–02 line, whereas only one LOH event was detected among asexual daughters of the CH‐H‐876 line (Table 1). Thus, genome‐wide rates of LOH, assessed bioinformatically, were calculated per locus per generation, yielding the estimates of 4.7 × 10^−3^ for IXF1, 3.1 × 10^−3^ for RM1–30, 3.7 × 10^−5^ for RM1–02 and 2.1 × 10^−5^ for the CH‐H‐876 line, respectively (Table 1).

**Figure 2:**
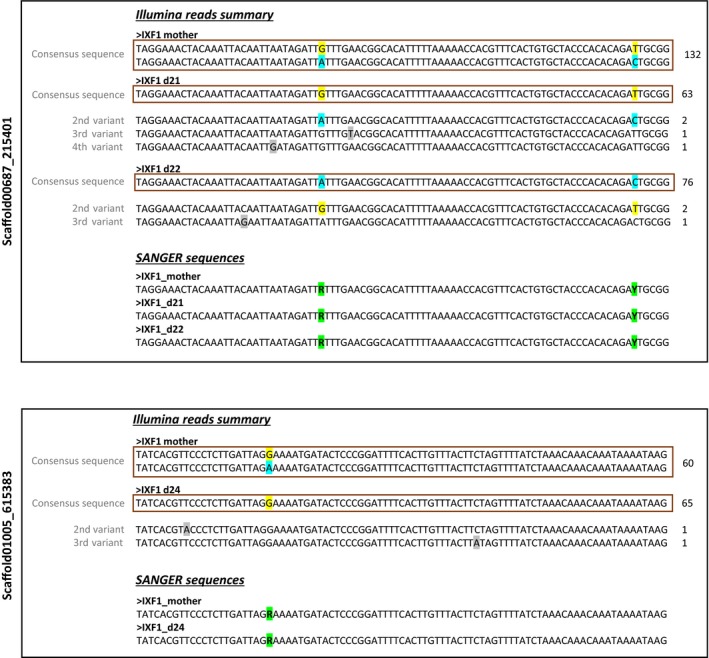
Examples of falsely annotated loss of heterozygosity (LOH) events. Detected Illumina short‐reads and the corresponding Sanger sequence for the stem mother (IXF1_mother) and the daughters showing LOH (IXF1_d21, IXF1_d22 and IXF1_d24) are depicted. For simplicity, only the heterozygous consensus sequence for one of two stem mother samples (see text) is shown, whereas all short‐read variants detected are shown for daughter individuals and the homozygous consensus sequence that passed our bioinformatic check is marked with the red frame. Maternal SNPs are labelled with yellow and blue squares, whereas the grey squares are marking the unrelated (sequencing error) nucleotide changes. Green squares are marking polymorphic sites detected in Sanger sequences. Numbers on the right are denoting the coverage for a locus/variant in each depicted individual. Upper panel is showing Illumina reads summary and Sanger sequences for the restriction site‐associated DNA (RAD)‐locus Scaffold00687_215401 indicating LOH in two parthenogenetic daughters (IXF1_d21 and IXF1_d22), whereas the lower panel is showing the same summary for the RAD‐locus Scaffold01005_615383 that appeared homozygous in one daughter (IXF1_d24) but presents a different type of erroneous call since the second maternal variant was not even sequenced at the low coverage

Our per locus LOH rates were similar to per locus error rates (Table 1). Hence, it is possible that the observed LOH events were actually caused by erroneous genotype calls. Moreover, instances showing LOH mainly fall into a lower quartile of the coverage distribution (average 49X, *SD* = 5.5) indicating that those were poorly covered loci or possible deletions causing hemizygosity. To test between these two scenarios, we used Sanger sequencing for examination of seventeen most promising loci showing LOH. These were our strongest candidates for LOH events, showing LOH in different lines, having high read coverage or showing LOH in markers flanking the same restriction site. In all seventeen instances, putative LOH events proved to be false positives; that is, presence of maternal polymorphism was confirmed in the re‐sequenced daughters (Figure 2).

## 4 DISCUSSION

Ameiotic recombination resulting in LOH may have important consequences for the evolutionary potential of asexual lineages. In this study, we performed reduced representation genome sequencing using the RAD‐sequencing protocol to address the occurrence of genome homogenization events within a single asexual generation of *D. magna*. This design allowed us to minimize possible selection against LOH and to exclude cryptic sexual events. We have assayed in total 174,270 RAD‐marker loci (on average 4,357 loci in each of the 40 asexual daughters) covering a total of 16.44 Mb of genomic sequence and including 310,932 heterozygous sites (1.8 heterozygous sites per RAD‐marker, on average). Still, we were not able to attest any LOH events in four independent genetic backgrounds (clonal lines) of *D magna*. Though a substantial number of LOH events were detected in two of four asexual lines with RAD‐sequencing (IXF1 and RM1–30), subsequent validation of putative LOH incidents by Sanger sequencing revealed these as false positives where heterozygotes had appeared as homozygotes. More precisely, maternal heterozygosity was confirmed in loci that were scored as homozygous in the RAD‐sequencing analysis of asexual daughters (Figure 2). This revealed that the LOH rate estimated from our RAD‐sequencing data most probably reflects sequencing biases that were not detectable using the bioinformatics analysis alone. Thus, the true rate of LOH must be substantially lower than our error‐inflated rate estimate.

Several possible sources of error in RAD‐sequencing could have caused allele dropouts that would appear as LOH in our data. These include restriction fragment length bias, stochastic events related to sequencing or PCR, and sequencing errors (Davey et al., 2013; Gautier et al., 2013). To minimize the systematic bias due to variation in restriction fragment length (Davey et al., 2013), we only considered informative loci (heterozygous in stem mothers) that were successfully genotyped in at least 80% of daughters, as markers for our analysis. Allele dropout due to mutations in a restriction enzyme recognition site is not very likely since this would result in a failure to cut the DNA at that location and the given allele would not be sequenced at all. However, in many instances the second variant of a heterozygous locus was detected but with a coverage of three or less and it was therefore indistinguishable from a sequencing error (Figure 2, upper panel). Taken together, this indicates that false homozygotes are primarily caused by preferential PCR amplification of one allele over the other (PCR duplicates) or stochastic events related to sequencing. More extensive analysis of the RAD‐sequencing data would be required to confirm the precise source of the genotyping error, which was beyond the scope of this paper. Nevertheless, our results strongly indicate that the future studies should be particularly cautions of potential errors induced by the chosen methodology, especially when searching for rare, discrete events such as LOH or mutations.

Previous studies using microsatellite markers in mutation‐accumulation lines of *D. pulex* and *D. obtusa* have estimated LOH to occur at a frequency that ranged from 10^−5^ to 10^−4^ per locus per generation (Omilian et al., 2006; Xu et al., 2011). Even though the RAD‐loci are rather different from microsatellite loci, if these rates would hold for our RAD‐loci, we should have detected between 1.7 and 17 true LOH events across all lines assayed (based on the total of 174,270 loci assessed). More recent assessments of LOH in *D. pulex* using whole‐genome sequencing obtained similar estimates of the LOH rate with values between 2.14 × 10^−5^ (Keith et al., 2015) and 4.82 × 10^−5^ per site per generation (Flynn et al., 2017). Since we investigated a total of 310,932 heterozygous sites (the RAD‐markers were heterozygous at 1.8 sites on average), this would translate into 7–15 LOH events expected in our dataset. In addition, all previous estimates are potentially confounded by selection against LOH that might have occurred during mutation‐accumulation lines, and hence, they may be considered minimum estimates. Nevertheless, we were unable to identify any true LOH events in our data, which suggests that LOH events were either absent or much less frequent than expected based on previous estimates.

Our inability to detect LOH could indicate that there is variation between *D. magna* and its related species *D. pulex* and *D. obtusa*, concerning the mechanism of ameiotic reproduction. And indeed, based on current data, it is difficult to infer what is the actual mechanism causing the LOH in *D. pulex*. In contrast to the previous assumption that LOH in adjacent microsatellite markers was caused by CO recombination (Omilian et al., 2006; Xu et al., 2011), Keith et al. (2015) used the whole‐genome sequencing to report the mean length of LOH tracts in *D. pulex* to be short (>250 bp) and, therefore, more likely caused by gene conversions. In addition, many LOH incidents were associated with large scale duplications typical for nonallelic homologous recombination (or ectopic gene conversions) that is invariably associated with repetitive (paralogous) sequences within the genome (Parks, Lawrence, & Raphael, 2015; Sasaki, Lange, & Keeney, 2010). Both *D. pulex* and *D. magna* genomes are extraordinarily rich in such repetitive sequences (Colbourne et al., 2011; *Daphnia* Genome Consortium). However, more recent study by Flynn et al. (2017), also using the whole‐genome sequencing approach, has reported a similar LOH rate but caused by a large LOH event, spanning 6 Mb of sequence (the entire linkage group) in one of the 23 mutation‐accumulation lines assayed. The authors propose that LOH in this case was due to ameiotic recombination (followed by internal deletions, (see Flynn et al., 2017)) rather than many gene conversions. In our study, we cannot exclude the possibility that nonreciprocal gene conversions occurred in genomic regions that were not covered by our markers (especially in repetitive genomic regions) or that a few LOH events detected bioinformatically are accurate homozygote calls. However, owing to a high‐density of RAD‐markers, with an average distance of 30 kb (as estimated based on a current genome assembly), we are confident that the exchange of long genomic tracts due to reciprocal CO recombination did not occur along the 400 chromosomes that were screened (10 chromosome pairs in 40 asexual daughters). Mapping of RAD‐markers to the third‐generation linkage map of *D. magna* (Dukić, Berner, Roesti, Haag, & Ebert, 2016) indicated that none of the putative LOH events occurred at neighbouring RAD‐markers in any of the asexual daughters, except for two cases of RAD‐markers flanking the same restriction site, which both turned out to be false positives during re‐sequencing. The 95% CI for the proportion when the observation is zero of 400 (a total of 400 chromosomes in 40 asexual daughters) extends from 0 to 0.0075 (McCracken & Looney, 2017). We can therefore conclude that the true rate of CO events is <1% (except perhaps for very close double COs within repetitive regions or terminal COs, which may go unnoticed in our data). Furthermore, our results suggest that future studies should probably concentrate on methods that accurately allow inference of short LOH events, even if they occur at low frequency.

Besides potential inter‐specific differences between *D. magna* and *D. pulex*, environmental factors might have also contributed to divergent LOH rates in the different *Daphnia* studies. Stressful conditions might elevate the rates of LOH by inducing DNA repair via homologous recombination and/or chromosome mis‐segregation, as previously demonstrated in *C. albicans* (Forche et al., 2011). Specifically, in our study, animals were kept individually and fed ab libitum to minimize potential stress‐induced LOH. There is no indication that *D. pulex* studies were carried out under particularly stressful conditions either; thus, we are reluctant to attribute the differences between studies to environmental stressors.

We have also tested whether the mother's age has any impact on generation of LOH due to genomic instability that is expected to occur with organismal senescence (Burhans & Weinberger, 2007), but we did not find any evidence for LOH in daughters produced by older females (the 12th clutch). Interestingly, in yeast it was shown that LOH in the offspring from young cells was caused by rare reciprocal CO recombination, although the rate of LOH was 40‐ to 200‐fold higher in the cells produced by old cells and it was mainly caused by nonreciprocal gene conversions (McMurray & Gottschling, 2003). Thus, our inability to detect elevation in LOH rates in older mothers could also be due to inherent limitations of the methodology employed in this study where we could have missed LOH caused by gene conversions or some other indication of genome instability, like genome rearrangements that are difficult to capture by sequencing in general. Alternatively, it is also possible that due to the nature of *Daphnia* life cycle, they have evolved better mechanisms to fight the consequences of genome deterioration due to ageing.

### 4.1 Implications for evolutionary biology

Clonality (with the exception of rare mutations) of asexual reproduction is a bedrock assumption in a great majority of models aiming to explain prevalence of sexual reproduction despite its high costs (Hartfield & Keightley, 2012; Kondrashov, 1993; West, Lively, & Read, 1999). However, since DSBs are an inevitable by‐product of cellular divisions (reviewed in Aguilera & Gómez‐González, 2008; Huertas, 2010), perfect clonality may be difficult to achieve within asexual lineages. In this light, clonality is merely a concept “dependent upon the resolving power of molecular markers” (Loxdale & Lushai, 2003) and some levels of homology‐based DSB repair leading to LOH are expected to occur. One model that takes the possibility of LOH during asexual reproduction into account is the “loss of complementation—LOC hypothesis” proposed by Archetti (2010, 2004a,2004b,). The basic logic of the LOC hypothesis is that the recombination processes causing LOH during asexual reproduction will lead to unmasking of recessive deleterious mutations (LOC). Consequently, the details of LOC will depend on the number of recessive deleterious mutations (lethal equivalents) and the proportion of the genome that becomes homozygous every asexual generation. As shown by Archetti, LOC may lead to a cost of asexual reproduction outweighing the two‐fold cost of sexual reproduction, but only under some combinations of parameters, including a sufficiently high LOH rate (Archetti, 2004b, 2010). However, our study together with the latest findings in *D. pulex* (Keith et al., 2015) indicates that long reciprocal allelic exchanges (COs) during parthenogenesis are less frequent than suggested by previous studies (Omilian et al., 2006; Xu et al., 2011), and that LOH may be restricted to short genomic regions. This implies that the portion of the genome experiencing LOH in a single asexual generation is too low to generate a high cost of asexuality due to LOC. Therefore, at least in *D. magna*, there is no support for the LOC hypothesis (Archetti, 2004b, 2010). For apomixis to bear high cost from increased homozygosity and the associated reduction in fitness, asexual lineages should harbour a large number of lethal equivalents. Even though this possibility cannot be excluded, it is not very plausible since it would make asexual lineages difficult to maintain in the laboratory, which is not the case. Nevertheless, possibility for LOH during asexual reproduction should not be ignored when discussing the evolutionary potential and the age of asexual lineages (Hartfield, Wright, & Agrawal, 2016) and more models accounting for this rare phenomenon are needed.

## CONFLICT OF INTEREST

All authors declare that they have no conflict of interest.

## Data Availability

Analysis scripts and processed data are available on Dryad (https://doi.org/10.5061/dryad.c9q4gt6). Raw sequencing data are available from the NCBI SRA database (PRJNA530020).
