# Supplementary material for: How clonal are clones? A quest for loss of heterozygosity during asexual reproduction in Daphnia magna
Source: J Evol Biol. 2019 Apr 10;32(6):619–28. doi: 10.1111/jeb.13443 (PMC6850383; doi:10.1111/jeb.13443)
Supplement: Supplementary file 1 [file JEB-32-619-s001.docx]

**Supplementary Table S1**. PCR primers for re-sequenced markers that were selected for verification of loss of heterozygosity events detected using RAD-sequencing. RAD-markers are named based on the alignment position of RAD-reads to the genome draft of *Daphnia magna* (v4.2)

| RAD_marker | Forward primer | Reverse primer |
| --- | --- | --- |
| scaffold00084_181078 | TTTTTGTGTGTGTGAAAGAGACC | CCTGGCAAGAAGAAAGAAGC |
| scaffold00024_965997 | TGGTGCCCTGACTGAGTGTA | TTTCCCATGTAACGACGACA |
| scaffold01005_1090145 | TTGAGGAAAGAGCGGGAATA | CACGGCCACAAAAATCTCAT |
| scaffold00687_215401 | CCCCAGATACCCGTACACAC | AGCTATCCAACGCGATCATT |
| scaffold01005_615383 | TTTTTGCTACCCCATGCAAT | CAAAGCCCCACAGCTATGAT |
| scaffold03258_520152 | TTTGTCCACTTTTCCGGTTC | CGTTATGAAGTGGACGCTGT |
| scaffold01654_178568 | GGCGGGTGTATAGCCAAGTA | AAAGAGACCGCGACTTTTGA |
| scaffold02723_2112 | TGAAGCGTGTTGCTTCTGTT | ATTGACATAGCCGCCAGATT |
| scaffold02581_769135 | AGGCCCTGATAGCATTACGA | GGAGACTCTGGAGCTGTTGG |
| scaffold02581_777742 | CAGGGCTTCCAGAATTACCA | CTGGAGCAAAAGGAGATGCT |
| scaffold01115_5223 | CCATTCTGATTGCGGTCTTT | CGTCCTTAATCCGACCACAT |
| scaffold00640_105924 | TCATCCTTGCTCTGGTCCTC | AATATGGCGACACAACATGC |
| scaffold03102_238072 | GGATTGCGTTAGGCAACAAA | GGCAACTGTGCCCTTGTATT |
| scaffold00512_163074 | GAATATCTGGACGCCATGCT | ACAATCAACAAATGCCGAAA |
